# Treatment with Ramucirumab-paclitaxel in a metastatic gastric cancer patient undergoing hemodialysis: A case report

**DOI:** 10.1097/MD.0000000000024795

**Published:** 2021-02-19

**Authors:** Min Joo Yang, Young Jin Choi, Hyo Jeong Kim, Do Young Kim, Young Mi Seol

**Affiliations:** Department of Hematology-oncology, Biomedical Research Institute, Pusan National University Hospital, Pusan National University School of Medicine, Busan, Republic of Korea.

**Keywords:** gastric cancer, hemodialysis, paclitaxel, ramucirumab

## Abstract

**Rationale:**

Ramucirumab, a human Ig 1 monoclonal antibody against vascular endothelial growth factor receptor-2, in combination with paclitaxel is a second-line chemotherapy for patients with metastatic gastric cancer. Several reports have suggested that dose adjustments of cetuximab, an anti- epidermal growth factor receptor antibody, are not required in patients with renal impairment. However, the combination chemotherapy of ramucirumab and cytotoxic drug for hemodialysis (HD) patients has not been reported.

**Patient concerns:**

A 65-year-old man on HD was diagnosed with gastric cancer and underwent a subtotal gastrectomy with D2 lymphadenectomy. Abdominal computed tomography (CT) was examined after completion of 8 cycles of adjuvant chemotherapy with capecitabine combination oxaliplatin.

**Diagnosis:**

The patient was diagnosed with advanced gastric cancer at stage IIIb (pT3N2M0) 11 months ago. Unfortunately, 9 months after the start of adjuvant chemotherapy, multiple liver metastases from gastric cancer were found by abdominal CT.

**Interventions:**

He began receiving weekly paclitaxel(80 mg/m^2^) and every 15-day ramucirumab (8 mg/kg). HD was performed next day after administration of chemotherapy and repeated 3 times a week.

**Outcomes:**

He was treated with ramucirumab without dose adjustment. The metastatic liver mass had a partial response, after 2 and 4 cycles of chemotherapy and had a stable disease up to 12 cycles of chemotherapy. No obvious adverse effect was observed during treatment. However, after 14 cycles chemotherapy, follow-up abdominal CT revealed progression disease of multiple liver metastasis and lymph nodes invasion.

**Lessons:**

The paclitaxel chemotherapy with ramucirumab is effective and safe in HD patients with metastatic gastric cancer. As seen in patients with normal kidney function, ramucirumab can be safely administered without a dose reduction.

## 1 Introduction

Gastric cancer is the fifth most common cancer and the third leading cause of cancer-related death and high mortality.^[1,2]^ Currently, chemotherapy based on a combination of fluoropyrimidines and platinum compounds is the standard treatment in first-line therapy.^[3]^ Based on the result of a randomized phase III trials, ramucirumab monotherapy or in combination with paclitaxel was proven safe and effective for patients with metastatic gastric cancer progressed after a first-line of chemotherapy.^[4,5]^

Ramucirumab is a human IgG 1 monoclonal antibody against vascular endothelial growth factor receptor-2 that prevents ligand binding and receptor-mediated pathway activation in endothelial cells, resulting in the inhibition of angiogenesis.^[6]^^.^

However, there is no data on ramucirumab therapy in patients undergoing chronic hemodialysis (HD). We herein report the first case of a metastatic gastric cancer patient on HD who was successfully treated with ramucirumab combination paclitaxel.

## 2 Case presentation

A 65-year-old man was undergoing peritoneal dialysis for chronic renal failure due to Focal segmental glomerulosclerosis 5 years previously and had switched to HD 3 times weekly beginning 3 years previously. He also had coexisting diseases, including hypertension. He complained of melena and hematochezia, and endoscopy identified a Her-2-negative poorly differentiated adenocarcinoma in the gastric body. He underwent subtotal gastrectomy with D2 lymphadenectomy, which led to a diagnosis of stage IIIb (pT3N2M0) gastric adenocarcinoma.

The patient started receiving chemotherapy in our hospital from May 2018. Initially, he received adjuvant chemotherapy with capecitabine 500 mg/m^2^ (50% of the standard dose of 1000 mg/m^2^) combination oxaliplatin 65 mg/m^2^ (50% of the standard dose of 130 mg/m^2^) every 3weeks. Chemotherapy was administered in the morning and he received HD on same day in the afternoon.

**Figure 1:**
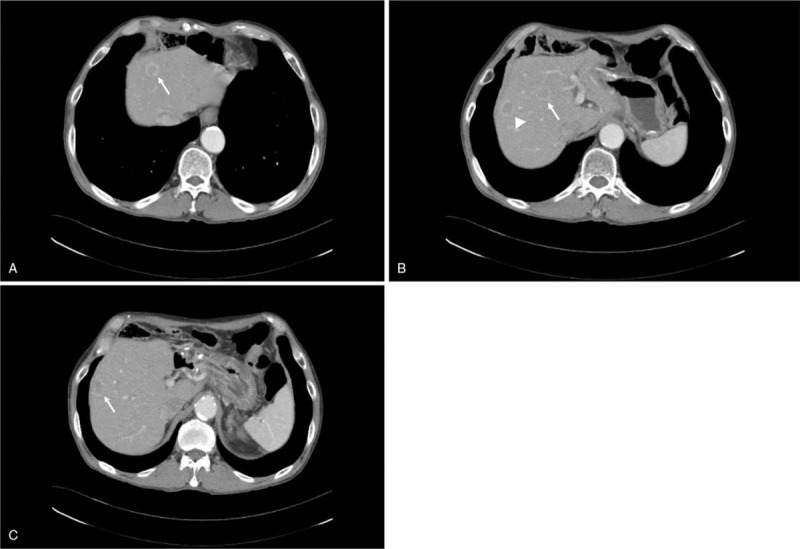
Abdominal computed tomography showed mutiple targetoid masses in both lobe of the liver (arrow). The largest 1 is 2.4 cm in S6 subcapsular area (arrow head).

However, after completion of 8 cycles of chemotherapy, abdominal computed tomography (CT) examination revealed multiple liver metastasis. (Fig. 1) In Feb 2019, for progressed disease, second-line chemotherapy was started. At that time, his Eastern Cooperative Oncology Group performance status, body surface area, and body weight was 1, 1.59m2, and 57 kg, respectively. He began receiving weekly paclitaxel (80 mg/m^2^) and every 15-day ramucirumab (8 mg/kg). HD was performed next day after administration of chemotherapy and repeated 3 times a week. Because the patient had HD at another hospital, renal monitoring and drug trough levels monitoring could not be confirmed.

**Figure 2:**
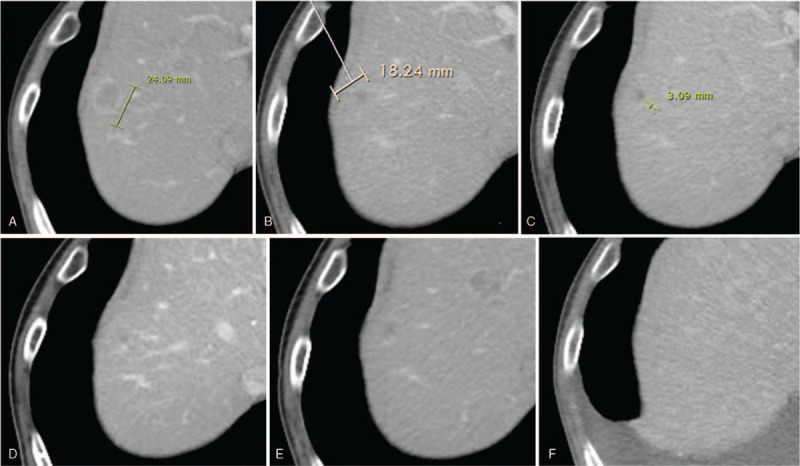
Computed tomography images show the liver metastasis. (A) At the start of chemotherapy; (B) after 2 cycles; (C) after 4 cycles; (D) after 6 cycles; (E) after 9 cycles; and (F) after 12 cycles of chemotherapy. While the liver metastatic mass expanded to 24 mm in diameter at beginning of chemotherapy, the mass reduced to subcentimeter sized (3 mm) after 4 cycles of chemotherapy.

On day 1 after cycle 2 chemotherapy, he developed grade 3 neutropenia (according to the National Cancer Institute Common Terminology Criteria for Adverse Events, version 4.0).^[7]^ The dose of paclitaxel was reduced up to 75% from the next cycle. After cycle 2 and cycle 4 of chemotherapy, abdominal CT was done to confirm the effectiveness of chemotherapy (Fig. 2A, B). While the liver metastatic mass expanded to 24 mm in diameter at beginning of chemotherapy, the mass reduced to 18 mm after 2 cycles of chemotherapy and subcentimeter sized (3 mm) after 4 cycles of chemotherapy. According to the Response Evaluation Criteria in Solid Tumor guidelines, we evaluated the patient as having a partial response.

For the sixth course of chemotherapy, because he developed grade 3 neutropenia despite the 25% reduction in dose, we further reduced the dose of paclitaxel on 60% and paclitaxel was skipped on days 8 from the next cycle in November 2019. After that, his metastatic liver tumor showed a stable disease, (Fig. 2 C–F) and he received 8 cycles of chemotherapy at that dose.

After cycle fourteen chemotherapy in July 2020, the progression of liver metastasis happened 16months after the second-line chemotherapy. Abdominal CT revealed progression disease of multiple liver metastasis and multiple small lymph nodes in the celiac axis, left gastric area, aortocaval area, left paraortic area. In August 2020, third-line chemotherapy was started with irinotecan (180 mg/m2). After cycle 1 chemotherapy, he was admitted to our department for neutropenic fever (ANC 80/uL), finally, he diagnosed enterocolitis and treated with antibiotics. After that, the third-line chemotherapy was stopped and he was received supportive care until October 2020. Since then, the patient has no longer visited our hospital.

## 3 Discussion

Recently, the phase 3 REGARD trial identified a new agent for second-line chemotherapy – ramucirumab, anti- vascular endothelial growth factor receptor-2 antibody.^[4]^ Follow up phase 3 RAINBOW trial ramucirumab plus paclitaxel significant extended second-line overall survival (OS; medican 9.6 vs 7.4 months; hazard ratio [HR] = 0.807; *P* = .017) and progression-free survival (PFS; medican 4.4 vs 2.9 months; HR = 0.635; *P* < .0001) over placebo-paclitaxel.^[5]^ Based on these results, we selected ramucirumab in combination with paclitaxel in second-line treatment for patients with advanced gastric cancer. Although the number of patients undergoing HD has been increasing to date, the guidelines for the management of chemotherapy in cancer patients undergoing HD have not yet been established. Renal dysfunction is not uncommon in cancer patients. However, patients with renal dysfunction are excluded from many clinical trials. Therefore, there is little data on the efficacy of all chemotherapy drugs in patients with renal impairment, and no recommendation for dosage and administration.

To the best of our knowledge, this is the first case of examining a combination of ramucirumab and paclitaxel for a patients undergoing HD.

Table 1 lists the published cases involving therapy with antiepidermal growth factor receptor antibody (ie, cetuximab, panitumumab) or anti VEGF antibody (ie, bevacizumab) in patients receiving HD.^[8–11]^

**Table 1:** Summary of data from previous case reports.

Cancer	Age	Gender	Regimen	Dose of anti-(V)EGFR antibody	Efficacy	Adverse events with anti-(V)EGFR antibody	Reference
Colorectal cancer	65	Male	mFOLFOX6 and bevacizumab	Full dose	Partial response	Not described	^[8]^
Colorectal cancer	71	Male	mFOLFOX6 and bevacizumab	Full dose	Stable disease	Not described	^[8]^
Colorectal cancer	71	Female	mFOLFOX6 and bevacizumab	Full dose	Progressive disease	Not described	^[8]^
Colorectal cancer	68	Male	Cetuximab and irinotecan	Full dose	Partial response	Not described	^[9]^
Colorectal cancer	66	Male	Cetuximab monotherapy	Full dose	Partial response	Not described	^[10]^
Colorectal cancer	62	Female	mFOLFOX6 and panitumumab	Full dose	Partial response	Grade 1 acneiform rash	^[11]^

The administration of ramucirumab in patients with renal impairment has not yet been reported, but pharmacokinetic behavior appears similar to cetuximab. The previous cases HD cancer patients treated with cetuximab suggested its safety, efficacy and no necessity for dose reduction.^[9,10]^ Another report analyzing the pharmacokinetics of cetuximab has shown that it can be safely used in patients with renal impairment without dose adjustment.^[12]^

Kobayashi et al^[11]^ showed that chemotherapy with panitumumab, a fully human IgG2 monoclonal antibody against epidermal growth factor receptor, is safe and effective in cancer patient with chronic kidney disease on HD.

Lisa O’brien et al^[13]^ reports about population pharmacokinetic meta-analysis of ramucirumab in cancer patients. This report showed that weight-normalized dosing regimen is appropriate for ramucirumab therapy, and dose adjustment was not required for patients with mild to moderate renal impairment or mild hepatic impairment. However, ramucirumab clearance in HD has not yet been analyzed.

In our case, ramucirumab was administered without a dose reduction and no side effects were observed. Although the mechanism is slightly different, ramucirumab can be safely used in patients with renal impairment, but further investigations involving drug clearance are required.

In terms of the efficacy of combined chemotherapy ramucirumab and paclitaxel, the mPFS and OS were 4.4 months and 9.6 months, respectively, in the RAINBOW trial.^[5]^ For our patient, the PFS was about 17 months, so we believe that combination chemotherapy with ramucirumab and paclitaxel is effective for patient undergoing HD compared to previous reports.

In conclusion, combination chemotherapy with ramucirumab was safe and effective in patients with chronic kidney disease undergoing HD, similar to that seen in patients with normal renal function. However, it is necessary to determine the dosage and administration through additional pharmacodynamic therapy.

## Author contributions

**Conceptualization:** Min Joo Yang, Young Mi Seol, Young Jin Choi, Hyo Jeong Kim, Do Young Kim.

**Investigation:** Min Joo Yang, Hyo Jeong Kim, Do Young Kim.

**Supervision:** Young Mi Seol, Young Jin Choi.

**Writing – original draft:** Min Joo Yang.

**Writing – review & editing:** Young Mi Seol.
